# Risk estimation of distant metastasis in node-negative, estrogen receptor-positive breast cancer patients using an RT-PCR based prognostic expression signature

**DOI:** 10.1186/1471-2407-8-339

**Published:** 2008-11-21

**Authors:** Andrew Tutt, Alice Wang, Charles Rowland, Cheryl Gillett, Kit Lau, Karen Chew, Hongyue Dai, Shirley Kwok, Kenneth Ryder, Henry Shu, Robert Springall, Paul Cane, Blair McCallie, Lauren Kam-Morgan, Steve Anderson, Horst Buerger, Joe Gray, James Bennington, Laura Esserman, Trevor Hastie, Samuel Broder, John Sninsky, Burkhard Brandt, Fred Waldman

**Affiliations:** 1 Breakthrough Breast Cancer Research Unit, King's College, London, UK; 2 Guy's Hospital, London, UK; 3 Celera, LLC, Alameda, CA, USA; 4 Guy's and St Thomas' Hospital Breast Research Tissue & Data Bank, London, UK; 5 Comprehensive Cancer Center, University of California San Francisco, San Francisco, CA, USA; 6 Rosetta Inpharmatics, A wholly owned subsidiary of Merck & Co. Inc., Seattle, WA, USA; 7 Cellular Pathology Department, St Thomas' Hospital, London, UK; 8 Laboratory Corporation of America, Triangle Park, NC, USA; 9 Institute of Tumor Biology, University Medical Center, University of Hamburg, Hamburg, Germany; 10 Life Sciences Division, Lawrence Berkeley National Laboratory, Berkeley, CA, USA; 11 Department of Pathology, California Pacific Medical Center, San Francisco, CA, USA; 12 Carol Franc Buck Breast Cancer Center, University of California San Francisco, San Francisco, CA, USA; 13 Departments of Statistics, and Health Research & Policy, Stanford University, Stanford, CA, USA

## Abstract

**Background:**

Given the large number of genes purported to be prognostic for breast cancer, it would be optimal if the genes identified are not confounded by the continuously changing systemic therapies. The aim of this study was to discover and validate a breast cancer prognostic expression signature for distant metastasis in untreated, early stage, lymph node-negative (N-) estrogen receptor-positive (ER+) patients with extensive follow-up times.

**Methods:**

197 genes previously associated with metastasis and ER status were profiled from 142 untreated breast cancer subjects. A "metastasis score" (MS) representing fourteen differentially expressed genes was developed and evaluated for its association with distant-metastasis-free survival (DMFS). Categorical risk classification was established from the continuous MS and further evaluated on an independent set of 279 untreated subjects. A third set of 45 subjects was tested to determine the prognostic performance of the MS in tamoxifen-treated women.

**Results:**

A 14-gene signature was found to be significantly associated (p < 0.05) with distant metastasis in a training set and subsequently in an independent validation set. In the validation set, the hazard ratios (HR) of the high risk compared to low risk groups were 4.02 (95% CI 1.91–8.44) for the endpoint of DMFS and 1.97 (95% CI 1.28 to 3.04) for overall survival after adjustment for age, tumor size and grade. The low and high MS risk groups had 10-year estimates (95% CI) of 96% (90–99%) and 72% (64–78%) respectively, for DMFS and 91% (84–95%) and 68% (61–75%), respectively for overall survival. Performance characteristics of the signature in the two sets were similar. Ki-67 labeling index (LI) was predictive for recurrent disease in the training set, but lost significance after adjustment for the expression signature. In a study of tamoxifen-treated patients, the HR for DMFS in high compared to low risk groups was 3.61 (95% CI 0.86–15.14).

**Conclusion:**

The 14-gene signature is significantly associated with risk of distant metastasis. The signature has a predominance of proliferation genes which have prognostic significance above that of Ki-67 LI and may aid in prioritizing future mechanistic studies and therapeutic interventions.

## Background

Despite considerable progress over past decades, breast cancer remains the most frequent major cancer and the second most common cause of cancer-death in women, with approximately half of new cases being estrogen-receptor positive and lymph-node negative. Recurrence at a distant site is a key driver of mortality from breast cancer.

Hormone receptor status provides critically important classification of outcome and clinical benefit from adjuvant endocrine therapies. However, future progress against breast cancer will depend, in part, on expanding knowledge regarding novel constellations of genes involved in the risk of micrometastatic spread and subsequent progression. It is optimal that, where possible, the prognostic effects of these genes are discovered in a context not confounded by the continuously changing field of systemic therapy. The use of untreated patients for discovery and validation permits unequivocal identification of prognostic genes not confounded with response genes, thereby permitting pathway directed therapies to be considered and allowing identification of those patients who might avoid the morbidity of adjuvant systemic therapy without significant risk of metastasis. For this reason, we studied formalin-fixed, paraffin-embedded (FFPE) tissue to identify a prognostic gene-expression signature in women with operable, invasive breast cancer that was estrogen receptor positive and lymph node negative and who received no systemic therapy following surgical resection of the primary tumor.

Recently, several expression signatures have been described for predicting distant metastatic risk for breast cancer, and assays derived from these signatures have shown a potential to improve prognostic accuracy, treatment choice, and disease outcomes in women diagnosed with early-stage breast cancer [1-12]. These signatures vary in the number of genes used, the types of tissues required (fresh frozen vs. paraffin-embedded), the technologies employed, and the platforms used. For example, MammaPrint [1-3], a DNA microarray assay that uses frozen tissue, is based on a 70-gene prognostic signature. The 21-gene Oncotype DX test [4-6] (16 cancer-related gene signature that includes the ER, PR and HER2 genes and 5 normalization genes), and the 2-gene ratio (HOXB13/IL17BR) test [7,8] are RT-PCR assays that use fixed tissues. Using a single data set, Fan et al [13] compared the predictions derived from 5 different gene signatures that included the 3 noted above and found substantial agreement in outcome classification in 4 out of 5 signatures with the 2-gene ratio test being the exception.

Given the large number of genes purported to be prognostic for breast cancer, we selected a subset of these genes to analyze on patients with N-, ER+ tumors. Our study is notable for the a) absence of systemic treatment, b) broad and representative breast cancer population tested, and c) long periods of follow-up. By combining genes primarily from the 70-gene signature, the 21-gene panel and from those reported by Dai et al [14] and West et al [15], we sought to find markers that could be further validated in untreated and treated study populations. In this report, we present a 14-gene signature that is associated with risk of distant metastasis. The signature was derived from untreated women, has been validated in an independent sample set of untreated women and was additionally evaluated in a sample set of tamoxifen-treated women. The gene-expression profile can be carried out with routinely fixed tissue on existing real-time PCR instruments for widespread testing and may permit more effective selection of conventional therapeutic anti-cancer agents, alone or in combination, for clinical trials.

## Methods

### Patients

#### Training set of untreated patients

The training set was derived from a cohort of 393 subjects accrued from 1975 to 1986 at the California Pacific Medical Center (CPMC) with a diagnosis of lymph node-negative T1 and T2 breast cancer. The primary study investigated the prognostic utility of tumor grade and Ki-67 labeling index in N- breast cancer. The patient population was largely untreated by systemic adjuvant therapy. All patients were followed for a minimum of 8 years or until death with a median follow-up time of 14.8 years. Tumors were classified according to WHO guidelines [16] and histological grade established using the modified Bloom and Richardson method [17]. The ER status of patient samples was determined by ESR1 ligand-binding methods. The use of patient material and data for this study has been approved by the institutional medical ethics committee.

We profiled 315 patients from whom sufficient amounts of amplifiable mRNA were extracted from formalin-fixed, paraffin-embedded tissues. Since ER status was missing for a large number of subjects (114 samples or 36%), we chose to reanalyze and standardize the ER results using mRNA measurements. Of the 315 patients, 106 subjects were excluded either because they received systemic therapy (N = 12) or because they were found to be ER-negative (N = 90) or both (N = 4), and 67 subjects met the inclusion criteria but were missing relapse clinical information, leaving 142 patients for further analysis.

#### Validation set of untreated patients

A retrospective search of the Breast Tissue and Data Bank at Guy's Hospital was made to identify an analogous cohort of patients diagnosed with primary breast cancer and who had definitive local therapy (breast conservation therapy or mastectomy) but without additional adjuvant systemic treatment. The study group was restricted to women diagnosed between 1975 and 2001, with a clinical tumor size of 3 cm or less, pathologically uninvolved auxiliary lymph nodes, ER+ tumor and with more than 5 years follow-up or recurrence or death prior to 5 years. Tumors were classified according to WHO guidelines [16] and histological grade established using the modified Bloom and Richardson method [17]. ER status on this group of patients had been determined using the standard IHC assay but we chose to reanalyze and standardize the ER results using mRNA measurements to be consistent with the training set (see Additional file 1 for concordance of ER status by IHC and RT-PCR). The standard Dako HercepTest method was used for scoring and defining positive/negative status. HER2 IHC testing was carried out on a Biogenex i6000 autostainer using Dako A0485 HER2 antibody (diluted 1:1000) with detection by Envision/HRP kit (Dako K5007). Cases with a HER2 score of 2+ or greater considered positive and 1+ or 0, negative.

A total of 415 patients were identified who also had sufficient FFPE tissue available for RNA extraction. From this group there was sufficient quantity and quality of mRNA to profile tumors from 303 patients. A further 24 cases were excluded from the study: 4 patients had bilateral breast cancer prior to distant metastasis, 6 had a missing gene expression value, 9 tumors proved to be ER-negative upon re-assessment using the mRNA expression assay, 3 were node positive and 2 were male patients. Thus, in total 279 patients were included in the analyses. The median follow-up time of the 279 patients was 15.6 years. The use of patient material and data for this study has been approved by Guy's Research Ethics Committee (04/Q0704/137).

#### Tamoxifen-treated patient study

A cohort of 45, N-, T1, ER+ patients who had received tamoxifen therapy and underwent surgery between 1990 and 1999 from the University of Muenster, Germany was used. The median follow-up time of the 45 patients was 5.8 years. The use of patient material and data for this study has been approved by the institutional medical ethics committee.

### Endpoints

We chose time from surgery to distant metastasis, also referred to as distant metastasis-free survival (DMFS), as the primary endpoint. Subjects were considered to have an event at the time of diagnosis of distant metastases or were censored at the earliest occurring date of contra-lateral recurrence, death without recurrence or last follow-up. The definition of DMFS endpoint, its events and censoring rules were aligned with those adopted by the National Surgical Adjuvant Breast and Bowel Project (NSABP) for the prognostic molecular marker studies [4]. We also analyzed the endpoint of overall survival (OS), which was defined as time from surgery to death from any cause.

### Sample processing

Five 10 μm sections of each paraffin block were used for RNA extraction. A macrodissection on the samples was performed to isolate RNA from the cancer cell areas which had been marked by a pathologist on a guide slide. Total RNA was extracted from the FFPE tissue sections using a modified commercially available isolation kit (Zymo Research, Orange, CA). Briefly, the FFPE section slides were deparaffinized in xylene, washed consecutively with 100%, 90%, and 70% ethanol, air dried at room temperature and the tissues transferred to a tube. Following digestion with proteinase K for 18 to 24 hours at 55°C, the samples were spun down and the supernatants transferred to new tubes. A mixture of 100% ethanol and extraction buffer was added to the supernatant and loaded onto Zymo-Spin II Columns. The columns were treated with a series of washes that include a DNase treatment step. Total RNA was eluted with TE buffer that was heated to 65°C.

### Determination of amplifiable RNA

The quality of the RNA extracted from formalin fixed tissues varies and depends on a variety of factors, including the fixation process used, age of the samples, and storage conditions. Additionally, the use of formaldehyde can cause extensive cross linking of tissue components. In many cases, only a small fraction of the recovered RNA can be amplified by RT-PCR. To determine the amount of amplifiable RNA, we quantified the expression level of an endogenous gene, NUP214, in each sample by comparing it to a serially diluted Universal Human Reference RNA standard (Stratagene, La Jolla, CA). Approximately 0.5 ng of amplifiable RNA was used to profile each gene.

### Gene selection

We selected 197 candidate genes (see Additional file 2) from the published literature that include the prognosis genes reported by van't Veer et al [1] and Dai et al [14], the gene signature for response in tamoxifen treated women reported by Paik et al (8), and the ER status genes reported by West et al [15]. In addition, three endogenous "housekeeping" genes (NUP214, PPIG, and SLU7) were included and used to normalize expression levels of the other genes (see Additional file 3).

### Message enrichment

The amount of PCR-amplifiable RNA from the training set was insufficient to profile all 197 genes. Consequently, RNAs from the training set were enriched by pre-amplification with the MessageAmpII aRNA amplification kit (Ambion, Austin, TX) whereas RNAs in the validation sets were used directly. To assess the effect of the enrichment, we profiled and compared the14 genes using 50 paired enriched and unenriched samples from the training set. Good correlation (R^2 ^= 0.9931) was observed between the metastasis scores generated with the enriched vs. unenriched samples (see Additional file 4).

### Gene expression profiling

A single-step RT-PCR with SYBR^® ^Green was used for gene expression profiling essentially as previously described [18]. The assays were performed on the Prism 7900 Real-Time PCR system using the following thermocycling parameters: 50°C for 2 minutes; 95°C for 1 minute; 60°C for 30 minutes; 95°C for 15 seconds and 60°C for 30 seconds for 42 cycles. A Universal Human Reference RNA control was amplified with the appropriate candidate gene for each run. All assays were performed in duplicate. PCR primers were designed to amplify all known splice-variants, and the size of the PCR product was designed to be shorter than 150 bp to accommodate degraded RNA in archived FFPE samples.

The relative changes in gene expression were calculated by the ΔΔCt method [19]. The expression of each of the genes was first normalized to three endogenous control (HSK) genes then further normalized to a calibrator, reference RNA pool (Universal human reference RNA, Stratagene, La Jolla, CA). The ΔΔCt values of 197 genes which gave acceptable expression levels were used for statistical analyses. In the validation set, we only profiled 3 normalization genes and the 14 cancer-related genes that were selected in the training set for the prognostic signature, and ESR1 gene for ER status determination.

### Estrogen receptor status by expression analysis

After developing an ER mRNA cutoff for estrogen receptor status on separate samples (Iverson et al, J Mol Diagn, in press) and demonstrating a high concordance with IHC determination in the training and validation sample sets, we chose to use ER expression as criteria for ER status for consistency between sample sets (see Additional file 1).

### Ki-67 Labeling Index (LI) in training set

The MIB-1 monoclonal antibody to Ki-67 (AMAC, Inc, Westbrook, ME) was used at 1:200 dilution in PBS. Following standard preparation of slides, staining was visualized using biotinylated anti-mouse (Vector Laboratories) and Strepavidin-horseradish peroxidase (Zymed Laboratories); DAB was used as the chromogen, and hematoxylin as the counterstain. The invasive cancer on a slide was reviewed for immunoreactivity. The slide was first scanned using a 10× objective, and regions with high labeling chosen for counting at high power (40×). The Ki-67 LI was calculated as the fraction of positively stained nuclei in at least 1000 invasive cancer cells from multiple high power fields. Tumors above the median labeling index were categorized as high Ki-67; those below the median labeling index, as low Ki-67.

### Statistical analyses

For gene selection in the training set, the expression levels of each gene were standardized to have mean zero and variance of one, and missing values were imputed using a nearest neighbor algorithm [20]. We used the semi-supervised principal component (SPC) method [21,22] available in the PAM software package [23] with a Cox regression model [24] for time to distant metastasis to each gene. The genes were ranked by their univariate Cox scores. The first principal component of the genes that reached a certain threshold of the univariate Cox score was computed and applied in a Cox model with the principal component as a single variable. Internal cross-validation was used to determine the optimal threshold to select genes to optimize the Cox score with the principal component of the expression of the selected genes. With this procedure, 37 genes were selected from the training set. The number of genes included in the prognosticator was further reduced by a regression of the supervised principal component on the expression values of the 37 genes (see Additional file 5) while imposing a constraint on the size of the regression coefficients. This procedure, known as the Lasso [25], resulted in a linear combination of the expression values of 14 genes (the remaining gene coefficients were effectively shrunk to zero) that provided a good approximation of the supervised principal component. For simplification, since the regression coefficients of the 14 genes were of similar magnitude (see Additional file 6), a summary score was calculated as the sum of the 14 ΔΔCT measurements for each subject, and since lower values of the score were associated with higher probability of metastasis, the final metastasis score (MS) for each subject is defined as the negative of the summary score. The creation of the MS is described in the Additional file 7.

Differences in patient characteristics were assessed with the Wilcoxon rank-sum or Kruskal-Wallis test for continuous or ordinal measures and with the chi-square test for discrete measures. Cox proportional hazards models were used to estimate hazard ratios and Wald tests of the coefficients from these models were used to assess statistical significance of the variables. The MS was modeled both as a continuous variable as well as in discrete groups of high (≥ -23.5) and low (< -23.5) risk, the cut-point of which was determined as the median MS in the training set. Other covariates included in the multivariable Cox models included years of age (at surgery), tumor size (cm), histologic grade of tumor. HER2 status had also been ascertained on the validation set using the FDA approved scoring method. These data were used as a covariate for an additional multivariable model in the validation set. Estimates of distant metastasis free and overall survival for the high and low MS groups were calculated with the method of Kaplan and Meier [26] and confidence intervals for point estimates of survival were calculated using the complementary log-log transformation [27]. The probability of distant metastasis in 5 years and 10 years for individual patients was calculated from the survivor function as estimated by an accelerated failure time model including the continuous MS as the independent variable and assuming the event times have a Weibull distribution [28]. Statistical analysis was performed with PAM [23], SAS software version 9.1 [29] and R software version 2.4.1 [30].

Time dependent receiver operator characteristic (ROC) curves and area under the curve (AUC) to predict distant metastases within 5 years and 10 years and death within 10 years were estimated using the method described by Heagerty [31] with nearest neighbor estimation of the bivariate distribution of time and continuous MS [32]. Sensitivity and specificity were estimated for each time of interest using the cut-point defining high and low MS risk groups. Approximate 95% confidence intervals for the various diagnostic summary measures were calculated based on the standard errors estimated from 500 bootstrap samples [33]. Ten year risk estimates for relapse and mortality based on the Adjuvant! Online calculator [34] were obtained for the patients in the untreated validation set and subsequently used to plot the 10-year time dependent ROC curves for visual comparison with the ROC curves based upon the MS.

## Results

### Patient characteristics

In order to obtain representative community-based untreated samples we chose a N- and ER + cohort design from similar hospital settings from US and UK. The characteristics of the subjects included in the training and validation sets are presented in Table 1. The patients in the training set were older and had a higher proportion low grade and small tumors than the patients in the validation set. In addition, subjects in the untreated validation set were followed for a longer period of time (median = 15.6 years) than those in the training set (median = 8.7 years) and the treated validation set (median = 6.3 years).

**Table 1:** Clinical and pathological characteristics of patients from the training and validation sets

	**Training**	**Validation**
**Characteristics**	**n = 142**	**n = 279**
	**n (%)**	**n (%)**
Age		
<40	9 (6.4)	20 (7.2)
40 – 49	18 (12.8)	74 (26.5)
50 – 59	28 (19.9)	77 (27.6)
60 – 69	48 (34.0)	73 (26.2)
≥ 70	38 (27.0)	35 (12.5)
Missing	1	0
Median	64 yrs (SD 12.6)	55 yrs (SD 11.7)
Min. – Max.	31 – 89 yrs	29 – 87 yrs
Tumor diameter		
≤ 2 cm	126 (94.0)	168 (60.2)
> 2 cm	8 (6.0)	111 (39.8)
Missing	8	0
Median	1.2 cm (SD 0.50)	2 cm (SD 0.85)
Min. – Max.	0.3 – 2.9 cm	0.0* – 3.0 cm
Tumor grade		
Grade 1	74 (53.2)	60 (21.5)
Grade 2	61 (43.9)	166 (59.5)
Grade 3	4 (2.9)	53 (19.0)
Missing	3	0
Stage		
I	126 (94.0)	168 (60.2)
IIA	8 (6.0)	111 (39.8)
Missing	8	0
HER2		
Negative	na	251 (93.0)
Positive	na	19 (7.0)
Missing		9
Surgery		
Breast conserving	na	112 (40.1)
Mastectomy	na	167 (59.9)
Radiotherapy		
Yes	na	115 (39.7)
No	na	175 (60.3)
Distant recurrence		
Yes	31 (21.8)	71 (25.5)
No	111 (78.2)	208 (74.6)
Death of all cause		
Yes	56 (39.4)	134 (48.0)
No	86 (60.6)	145 (52.0)
Median follow up	8.7 yrs	15.6 yrs

*tumors were impalpable

To assess potential selection bias in the training set we compared the selected 142 patients to 67 patients who met the entry criteria but were not included because of missing clinical information. No significant differences were found between the included and excluded subjects with respect to age (median = 64.0 and 63.7 respectively; p = 0.51) or tumor size (median = 1.28 cm and 1.23 cm respectively; p = 0.39). However, tumor grade tended to be higher among the included subjects than the excluded subjects (47% and 32% with ≥ Grade 2 tumors respectively; p = 0.02) (see Additional file 8). Similarly, in the untreated validation set we compared the selected 279 patients to 118 patients who met the entry criteria but were not included due to insufficient RNA (n = 112) or missing RNA expression values (n = 6) for one or more of the 14 signature genes. No significant differences between included and excluded patients were found with respect to age (median = 56.4 and 55.6 respectively, p = 0.47), or tumor grade (71% and 78% with Grade 2 tumors respectively, p = 0.18) (see Additional file 8). Although median tumor size was 2 cm for both included and excluded patients, a test comparing the distributions of ordered ranks for the two groups indicated a significant difference (p = 0.02). Upon further inspection, we found a somewhat larger proportion of patients with tumor size greater than 2 cm among the included patients than among the excluded patients (40% vs. 31% respectively).

### Association of the 14-gene signature with DMFS and overall survival in the training set from CPMC

The median MS in the training set was -23.5 and ranged from -72.8 to 16.8. The MS performed well in the training set which is expected given that the outcome of subjects in this sample set was used to select the genes. Each unit increase in the MS was associated with a 4.2% increase (p = 0.0001) in risk of distant metastasis and a 2.4% increase (p = 0.003) in risk of death. The hazard ratio (HR), after adjusting for age, tumor size and tumor grade, comparing subjects above the median MS to subjects below the median MS was 3.2 (95% CI 1.27 to 7.87; p-value = 0.014) for risk of distant metastasis (Table 2) and 2.0 (95% CI 1.05 to 3.83; p-value = 0.036) for risk of death (Table 3). However, since these estimates of risk are likely biased due to the selection of genes from this same training set we next tested the MS in an independent validation set.

**Table 2:** Univariate and multivariate Cox proportional analyses of distant-metastasis-free survival

		Univariate analysis		Multivariate analysis	
			
Study	Variable	Hazard ratio (95% CI)	p-value	Hazard ratio (95% CI)	p-value
Training set (untreated set)	14-gene signature	4.34 (1.86–10.1)	0.001	3.16 (1.27–7.87)	0.014
	Age	1.00 (0.97–1.03)	0.853	1.00 (0.97–1.03)	0.956
	Tumor size	1.91 (1.00–3.63)	0.050	1.41 (0.68–2.91)	0.353
	Grade 2	2.21 (1.05–4.70)	0.038	1.45 (0.65–3.22)	0.360
	Grade 3	1.72 (0.22–13.3)	0.604	0.72 (0.09–6.00)	0.759

Validation set (untreated set)	14-gene signature	4.71 (2.33–9.51)	<0.0001	4.02 (1.91–8.44)	0.0002
	Age	1.03 (1.00–1.05)	0.024	1.02 (0.99–1.04)	0.193
	Tumor size	1.75 (1.25–2.46)	0.001	1.37 (0.95–2.00)	0.092
	Grade 2	2.43 (1.10–5.40)	0.029	1.25 (0.55–2.87)	0.592
	Grade 3	3.01 (1.23–7.39)	0.016	1.11 (0.43–2.87)	0.833

Tam-treated set	14-gene signature	3.61 (0.86–15.1)	0.079	3.50 (0.58–21.2)	0.172
	Age	1.04 (0.98–1.10)	0.241	1.06 (0.98–1.14)	0.166
	Tumor grade	1.50 (0.50–4.49)	0.468	NA	NA

**Table 3:** Univariate and multivariate Cox proportional analyses of overall survival

		Univariate analysis		Multivariate analysis	
			
Study	Variable	Hazard ratio (95% CI)	p-value	Hazard ratio (95% CI)	p-value
Training set (untreated set)	14-gene signature	2.48 (1.42–4.32)	0.001	2.00 (1.05–3.83)	0.036
	Age	1.08 (1.05–1.11)	<0.0001	1.08 (1.05–1.11)	<.0001
	Tumor size	1.42 (0.85–2.39)	0.181	1.00 (0.56–1.78)	0.994
	Grade 2	1.41 (0.83–2.40)	0.210	1.44 (0.80–2.59)	0.223
	Grade 3	0.80 (0.11–5.93)	0.830	1.15 (0.15–9.08)	0.897

Validation set (untreated set)	14-gene signature	2.26 (1.51–3.38)	<0.0001	1.97 (1.28–3.04)	0.002
	Age	1.06 (1.05–1.08)	<0.0001	1.06 (1.04–1.08)	<.0001
	Tumor size	1.61 (1.27–2.03)	<0.0001	1.07 (0.84–1.38)	0.571
	Grade 2	2.42 (1.35–4.34)	0.003	1.47 (0.80–2.70)	0.218
	Grade 3	2.95 (1.54–5.63)	0.001	1.32 (0.65–2.66)	0.442

### Validation of the 14-gene prognostic signature in samples from Guy's Hospital

**Figure 1:**
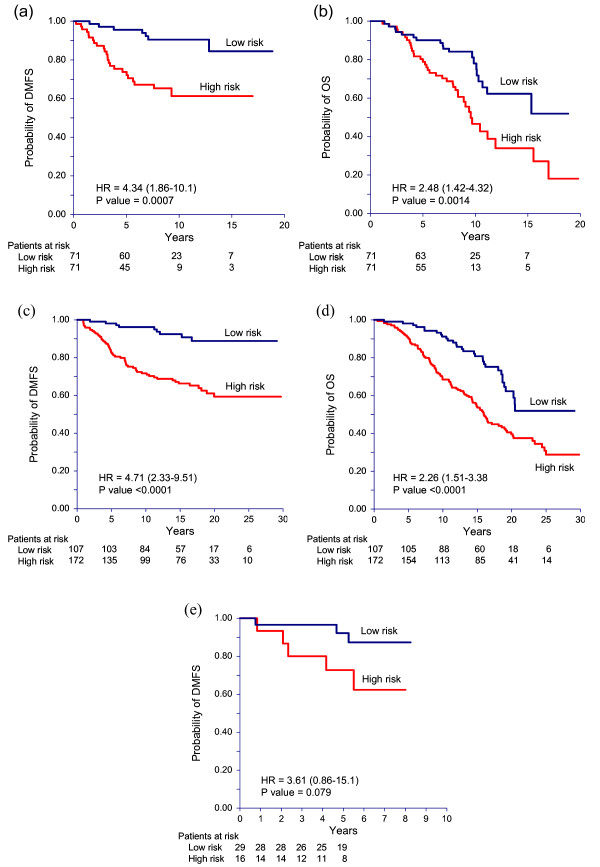
**Kaplan-Meier analysis for distant-metastasis-free survival (DMFS) and overall survival (OS).** a) DMFS of training set, b) OS of training set, c) DMFS of validation set, d) OS of validation set, e) DMFS of tamoxifen-treated set.

The MS was also associated with DMFS (p < 0.0001) and overall survival (p = 0.0004) when tested in the validation sample set from Guy's Hospital. Each unit increase of the MS resulted in an estimated 4.9% (95% CI 1.03 to 1.07) increase in risk of DMFS and a 2.4% (95% CI 1.01 to 1.04) increase in risk of death. Applying the identical cutpoint (< -23.5 vs. ≥ -23.5) as used in the training set resulted in 107 patients designated as low risk and 172 as high risk. In univariate analysis, the HR for the high risk group compared to the low risk group was 4.71 (95% CI 2.33 to 9.51, p < 0.0001) for DMFS (Table 2) and 2.26 (95% CI 1.51 to 3.38, p < 0.0001) for overall survival (Table 3). After adjustment for age, tumor size and tumor grade in Cox multivariate analysis the HR for the high versus low risk group were 4.02 (95% CI 1.91 to 8.44, p = 0.0002) and 1.97 (95% CI 1.28 to 3.04, p = 0.002) for DMFS (Table 2) and overall survival (Table 3), respectively. Among subjects in the high risk group, the estimated rates of DMFS were 83% (95% CI 76 to 88%) at five and 72% (95% CI 64 to 78%) at ten years of follow-up as compared to rates of 98% (95% CI 93 to 100%) and 96% (95% CI 90 to 99%) at five and ten years respectively among subjects in the low risk group (Figure 1). Rates of overall survival were 91% (95% CI 85 to 94%) at five years and 68% (95 CI 61% to 75%) at ten years among high risk subjects compared to 98% (95% CI 93 to 100%) and 91% (95% CI 84 to 95%) at five and ten years respectively among low risk subjects (Figure 1). The HR for the high versus low risk group did not differ significantly (p > 0.05) by age group (≤ 55, > 55) or by tumor grade (1, 2 or 3) for either the DMFS or overall survival endpoints. The HR for the endpoint of overall survival was 3.52 (95% CI 1.83 to 6.75) in subjects with tumor diameters of 2 cm or less compared to 1.23 (95% CI 0.70 to 2.17) in subjects with tumor diameters larger than 2 cm and this difference in hazard ratios was significant (p-value for interaction = 0.012). A similar trend, though not significant (p-value for interaction = 0.14), was seen for the endpoint of DMFS where the HR was 7.59 (95% CI 2.07 to 27.8) in subjects with small tumors (≤ 2 cm) and 2.53 (95% CI 1.05 to 6.12) in subjects with larger tumors (> 2 cm). The risk estimates of the high versus low risk group remained significant (p = 0.0002 for DMFS; p = 0.002 for overall survival) and were essentially unchanged (HR = 4.16 for DMFS; HR = 2.05 for overall survival) in additional multivariate models that included HER2 expression as a covariate although large changes would be unexpected due to the small number of HER2 positive subjects (N = 19).

### MS as a continuous predictor of probability of distant-metastasis-free survival

**Figure 2:**
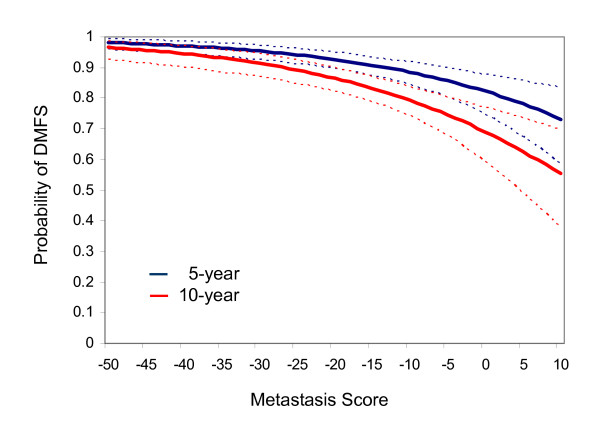
Metastasis Score as a continuous predictor of probability of distant- metastasis-free survival (DMFS) (5 and 10 year estimates with dashed lines as 95% CI).

While the threshold for determining the high and low risk groups based on the median value of the MS in the training set resulted in significant differences in DMFS estimates for these groups in the validation set, the risk for individual patients may be of a more continuous nature. Figure 2 shows the estimated probabilities of DMFS at 5 and 10 years based on a parametric regression model of the event times among the validation set subjects. Using this model, the 5 and 10 year probabilities of DMFS are 94.7% and 90.2% respectively for subjects with MS scores equal to the 25^th ^percentile in the validation set (MS = -27.4), 91.7% and 84.8% respectively for subjects with MS score equal to the median (MS = -17.3) and 88.0% and 78.5% respectively for subjects with MS score equal to the 75^th ^percentile (MS = -9.1).

### Diagnostic accuracy and predictive values

The sensitivity and specificity of the MS high and low risk groups to predict distant metastases were 96% (95% CI 89 to 100%) and 43% (95% CI 37 to 49%) respectively at 5 years and 93% (95% CI 87 to 100%) and 46% (95% CI 39 to 53%) respectively at 10 years. Sensitivity and specificity of the MS risk groups to predict death from any cause at 10 years were 84% (95% CI 75 to 94%) and 45% (95% CI 39 to 52%) respectively.

**Figure 3:**
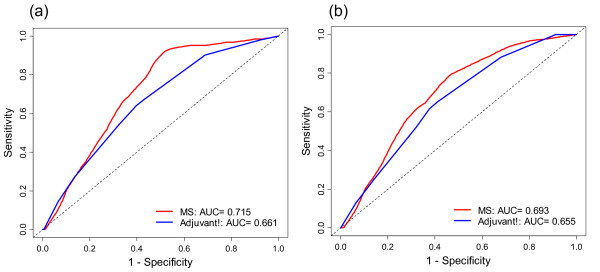
**Receiver operating characteristic (ROC) curves of 14-gene signature and of Adjuvants! software.** ROC curves for distant metastasis (a) and for death (b) within 10 years.

ROC curves of continuous MS to predict distant metastasis within 5 years and 10 years had AUC (95% CI) of 0.74 (0.66 to 0.81), and 0.71 (0.65 to 0.78). A ROC curve to predict death in 10 years had an AUC of 0.69 (0.62 to 0.77). Hence, MS are predictive of both distant metastases and deaths. Visual comparison of the MS and Adjuvant! Online ROC curves (Figure 3) for distant metastasis and overall survival at 10 years indicate the MS may provide additional diagnostic value, particularly for the distant metastasis endpoint.

### 14-gene signature in study with tamoxifen treated patients

In the study of 45 tamoxifen-treated patients (see Additional file 9 for clinical and pathological characteristics of the patients), each unit increase in the MS resulted in an estimated 4.9 percent increase in the hazard for DMFS although this association did not reach statistical significance (p = 0.085). Sixteen patients were classified as high-risk and 29 were classified as low-risk by MS using the cut point of -23.5. In univariate analysis, the HR for DMFS comparing high risk to low risk subjects was 3.61 (95% CI 0.86 to 15.1, p = 0.079) (Table 2). In multivariate analysis the HR was 3.50 (95% CI 0.58 to 21.2, p = 0.172). Five-year distant-metastasis-free survival rates were 73% (95% CI 40 to 89%) in the high risk group and 92% (95% CI 72 to 98%) in the low risk group (Figure 1).

### Comparison of metastatic expression signature and Ki-67 LI in training set

Using the median as the cutpoint for the Ki-67 LI, univariate analysis indicated this proliferation marker is significantly associated with DMFS (HR = 2.4, (95% CI 1.12 to 5.11, p = 0.02) and overall survival (HR = 1.71, (95% CI 1.00 to 2.93, p = 0.05) but is no longer significant after adjustment for MS in a multivariate analysis. The HR for Ki-67 were reduced to 1.55 (95%CI 0.69 to 3.45, p = 0.29) and 1.32 (95% CI 0.75 to 2.33, p = 0.33) for DMFS and overall survival respectively after adjustment for MS while the MS retained significance when adjusted for Ki-67 LI; the HR for the high versus low risk group were 3.64 (95% CI 1.49 to 8.90, p = 0.005) and 2.21 (95% CI 1.23 to 3.97, p = 0.008) for DMFS and overall survival respectively (Table 4). Moreover, the correlation coefficient, R^2^, between Ki-67 LI and MS was 0.30. We did not find a strong correlation (R^2 ^= 0.18) between Li-67 LI and the mRNA level of this gene (see Additional file 10).

**Table 4:** Univariate and multivariate analyses of 14-gene signature and Ki-67 LI for distant-metastasis-free survival (DMFS) and overall survival (OS)

		Univariate analysis		Multivariate analysis	
			
Endpoint	Variable	Hazard ratio (95% CI)	p-value	Hazard ratio (95% CI)	p-value
DMFS	14-gene signature	4.34 (1.86–10.1)	0.001	3.64 (1.49–8.90)	0.005
	Ki-67 LI	2.40 (1.12–5.11)	0.024	1.55 (0.69–3.45)	0.286

OS	14-gene signature	2.48 (1.42–4.32)	0.001	2.21 (1.23–3.97)	0.008
	Ki-67 LI	1.71 (1.00–2.93)	0.049	1.32 (0.75–2.33)	0.332

### Ontology of genes in the prognostic signature

A list of the 14 genes and overlaps with previously reported signatures is shown in Table 5. Subsets of genes of the previously reported signatures appear in the 14-gene signature: 9 genes overlap with the cell proliferation signature identified by Dai et al [14]; 2 genes overlap with the 21-gene panel by Paik et al [4], and 6 genes overlap with 70-gene panel by van't Veer et al [1]. The gene function analysis revealed that BUB1, CCNB1, MYBL2, PKMYT1, RACGAP1, CENPA, ORC6L, TK1, DIAPH3, RFC4, MELK, and RFC4 are associated with one or more functions of cell cycle control, apoptosis or DNA recombination and repair. UBE2S is a gene involved in ubiquitination. There are two genes, PRR11 and DC13, whose functions are not known. Pathway analyses revealed that the 14 genes in our prognostic signature are involved in a variety of biological functions, but a majority of genes are involved with cell proliferation. Ten of the 14 genes are associated with the TP53 pathways which have been found to be coordinately over-expressed in tumors of poor outcome.

**Table 5:** Gene name, description, and function of the 14-gene prognostic signature and the overlap with previously reported signatures

**Gene**	**Paik *et al***	**van't Veer *et al***	**Dai *et al***	**Gene Description**	**Gene Function**
BUB1			x	BUB1 budding uninhibited by benzimidazoles 1 homolog (yeast)	cell cycle

CCNB1	x			cyclin B1	regulation of cell cycle

CENPA		x	x	centromere protein A, 17kDa	chromosome organization and biogenesis (sensu Eukarya), nucleosome assembly

DC13		x		DC13 protein	assembly of cytochrome oxidase

DIAPH3		x	x	diaphanous homolog 3 (Drosophila)	actin cytoskeleton organization and biogenesis

MELK		x		maternal embryonic leucine zipper kinase	protein amino acid phosphorylation, ATP binding

MYBL2	x			v-myb myeloblastosis viral oncogene homolog (avian)-like 2	anti-apoptosis, regulation of cell cycle, development

ORC6L		x	x	origin recognition complex, subunit 6 homolog-like (yeast)	DNA replication

PKMYT1			x	membrane-associated tyrosine- and threonine-specific cdc2-inhibitory kinase	protein amino acid phosphorylation, regulation of cyclin dependent protein kinase activity

PRR11			x	proline rich 11	E2F transcription factor taget gene, regulation of cell cycle

RACGAP1			x	Rac GTPase activating protein 1	electron transport

RFC4		x		replication factor C (activator 1) 4, 37kDa	DNA replication

TK1			x	thymidine kinase 1, soluble	DNA metabolism

UBE2S			x	ubiquitin-conjugating enzyme E2S	ubiquitin cycle

## Discussion

Even though several breast cancer prognostic signatures have been published, the study described here is notable for several reasons. The use of untreated patients for the training and test sample sets permits unequivocal identification of prognostic genes that are not confounded with response genes, thereby providing insight into pathway directed therapies and opportunities for basic research. The prognostic signature does not contain ER, ER-responsive genes or HER2 and therefore circumvents the expressed concern that expression signatures should provide information independent of these valuable and routinely tested IHC markers. In addition, we have shown that the signature provides additional information than the commonly used Ki67 proliferation marker. This signature is expected to be generalizable given the consistent results observed in the geographically diverse sample sets. Our results further suggest that the prognostic score from untreated patients retains its prognostic value in tamoxifen-treated patients. The relatively small number of genes in the described signature will facilitate follow up functional studies in support of their mechanistic role in distant metastasis. Finally, the relatively small number of genes in this prognostic signature, which does not depend on a complex algorithm, coupled with the wide-spread use of fixed tissue and familiarity of RT-PCR should facilitate the broader transfer of these types of analyses to multiple testing laboratories as well as facilitate submission of in vitro diagnostic products to regulatory agencies.

We selected genes from 3 previously reported prognostic gene signatures plus ER-related genes and analyzed the expression of 197 genes in a training set of non-systemically treated, N-, T1/T2 (≤ 3 cm), ER+, breast cancer patients. A subset of 14 genes, found to be prognostic for breast cancer, was used to generate a metastasis score (MS) to quantify risk for individuals at different timeframes as well as dichotomize samples into high and low risk groups. Following initial selection and analysis within the training set, we validated the expression signature on an independent sample set using the precise dichotomized cutoff of the training set. Performance characteristics of the signature in the training and validation sets were similar. Univariate and multivariate hazard ratios to predict DMFS were 4.34 and 3.16 in the training set and 4.71 and 4.02 for the validation set, respectively. In multivariate analysis, only the metastasis score remained significant. The 14-gene prognostic signature also predicts overall survival with univariate and multivariate hazard ratios of 2.48 and 2.00 in the training and 2.26 and 1.97 in the validation set, respectively. When comparing the predictive accuracy with a commonly used Adjuvant! Online, the areas under the ROC curves were slightly higher for the 14-gene signature classification than for the Adjuvant! classification indicating MS may provide additional diagnostic value.

We were curious whether the signature developed in patients without systemic therapy would be predictive in tamoxifen-treated patients. In a study of a small number of tamoxifen-treated women, the signature predicted two risk groups using the same single cutpoint as for untreated patients, but the results only trended to significance due likely to sample size. Since tamoxifen treatment only reduces distant recurrence by approximately 30%, larger data sets will be required to discern the prognostic nature of the signature in women who do and do not respond to tamoxifen.

Several investigators [35,36] have queried whether molecular expression scores provide discrete information to those routinely provided by single or composite pathological prognostic tests already routinely provided. As an example, Ki-67 LI determined proliferation status has been reported in numerous individual studies as well as a meta-analysis study to be a prognostic factor for recurrence-free and disease-specific survival [37-44]. We tested Ki-67 LI because of the strength of reports in literature and availability in the training set. Ki-67 labeling index was predictive for recurrent disease; however, after adjustment for the metastatic expression signature this often used marker lost significance. As with two previous reports (Potemski et al [42] and Tan et al [43]) we did not find a strong correlation between the Ki-67 LI full range of staining and the mRNA levels of this gene.

The 14 upregulated genes represent a unique signature and do not fully overlap with any of the original 3 signatures from which the genes were selected. Three proliferation genes (BUB1, CCNB1 and MYBL2) highlighted in Whitfield et al [45] appear in the 14 gene signature described here but only MYBL2 overlaps with the p53 status signature recently reported by Miller at al [46]. Even though the TP53 genes have not appeared in lists of proliferation genes, network analysis of the genes of the proliferation signature described here is suggestive of network involvement (see Additional file 11). The signature lacks the ER and PgR genes. The absence of these hormonal receptors is not unexpected given that these genes have been reported to be weakly prognostic in untreated patients. The majority of the genes in the signature are involved in processes associated with tumor growth such as DNA replication (BUB1, CCNB1, CENPA, ORC6L, RFC4, TK1), cell cycle control (BUB1, CCNB1, MYBL2, ORC6L, PKMYT1, RACGAP1), cellular assembly and organization (BUB1, CCNB1, CENPA, DIAPH3), and ubiquitination (UBE2S). Many of the genes in the signature have been implicated in cancers. The known and inferred role of these genes in cell proliferation is consistent with their contribution to the disease process. While the 14-gene tumor expression profile reported here has practical importance in classifying distant metastasis as an outcome in patients with operable, invasive breast cancer, the identification of prognostically relevant gene pathways has ramifications for targeted therapy in the future, with applications to conventional cytotoxic drugs and novel experimental therapies [47-49].

The sample population and the experimental approaches we employed vary in some aspects from previously reported studies. First, the signature was developed and validated on FFPE samples from non-systemically treated breast cancer patients to capture solely prognostic information without confounding by genes that may play a role in recurrence and/or response to treatment. In contrast, Oncotype Dx [4] was trained in tamoxifen-treated patient samples – which may have contributed to the identification of ER and PgR as important markers. As discussed by Hayes [50], ER and ER-related genes are known to be positive predictors of endocrine therapy but only weakly prognostic. Second, our study population has a broad distribution of age covering both pre- and postmenopausal women that is representative of a typical breast cancer patient population. In comparison, the MammaPrint signature [1,2] was developed using samples from primarily younger women and the Oncotype DX signature [4] was developed using clinical trial samples. Third, the number and equal weighting of each of the genes of the signature permits more focused follow-up mechanistic studies. Fourth, the long duration of follow-up in the validation set allows quantification of risk over different time frames as well as categorizing risk into different groups. This is important as individuals differ substantially in their risk tolerance and time horizon concern. Fifth, the signature was developed on FFPE samples and expression analysis was performed using RT-PCR. This sample type enables analysis of archived sections that have extended outcome data as well as present day specimens that are routinely processed in a similar manner. Gene signatures developed on frozen tissues (for example, MammaPrint and wound response signatures [12]) would require a change in present sample collection and storage. Finally, clinical data reported by Esteva [51] suggest that a multigene expression profile assay, trained on tamoxifen treated samples, may not necessarily classify the risk of recurrent disease in patients with N(-) breast cancer who do not receive adjuvant tamoxifen or chemotherapy. The 14-gene prognostic signature reported here was developed on untreated patient samples, and as suggested by one of the referees, one potential implication of the current study is that the 14-gene expression signature may identify a low-risk patient-group with hormone receptor-positive breast cancer, whose predicted absolute survival benefit from systemic adjuvant therapy is so low that a woman, armed with this prognostic information, may favor the avoidance of the occasionally troublesome side effects of endocrine therapy.

The reported study has limitations. In order to identify a cohort of non-systemically treated patients, it was necessary to assemble samples from patients before tamoxifen became a routine treatment option. As a result, the samples in this cohort may not represent ER+ breast cancer patients today. In this study, we used a retrospective population-based cohort study design. While a cohort study is expected to have fewer hidden confounders and biases than a case-control study, we cannot exclude the presence of masked bias. Further, population-based cohorts have less uniformity than patients from the controlled setting of clinical trials. On the other hand, such studies are likely to be more representative of a community setting in which the molecular prognostic assay would be applied [52].

## Conclusion

In conclusion, we have identified and validated a gene expression signature, applicable to the analysis of routinely acquired FFPE tissue that adds important baseline prognostic information to assist women in their decisions about the size of absolute benefits and risks of adjuvant systemic therapy. The described signature does not include the modestly prognostic hormone receptor and HER2 markers and remains prognostic when proliferation, assessed by Ki-67 IHC score is included in the analysis, thereby simplifying interpretation of the resulting score relative to these standardly used markers. This report extends previous studies that identified genes involved in proliferation as important prognostic members of individual risk factors and combined signatures and highlights the implication of consistent biological and clinical associations with gene expression profiles. The limited number of genes in the signature will facilitate mechanistic studies and may serve as targets for future therapies. Further, the relatively small number of genes in this prognostic signature coupled with the familiarity of RT-PCR should facilitate the broader transfer of these types of analyses to multiple testing laboratories as well as facilitate submission of in vitro diagnostic products to regulatory agencies, both of which will result in expanded cost-effective access relative to laboratory developed assays offered by single laboratories.

## Competing interests

JJS, AW, CR, SK, KL and SB are employees of Celera Corp; SA and LK-M are employees of LabCorp.

## Authors' contributions

AT, FW, JS, BB, JG, SA and LE conceived the study. AW, KL, SB, CR and JS participated in the study design and data analysis. CG, RS, KC, PC, HB and JB provided clinical samples and background. KL, TH, KR and CR contributed to the statistical analysis. AW and SK supervised the sample preparation and RNA enrichment; HS, BM, and LK-M performed the RNA extraction. HD provided cancer gene list prior to publication. All authors have read and approved the final manuscript.

## Pre-publication history

The pre-publication history for this paper can be accessed here:



## Supplementary Material

Additional file 1 **Concordance for ER status by immunhistochemistry and RT-PCR.** We evaluated the concordance of ER status determined by RT-PCR and IHC for the Guy's untreated patients. Of the 287 patients, 97.6% and 96.9% were ER-positive by IHC and RT-PCR assay, respectively. The cutoff point for ER positivity of RT-PCR assay was prespecified as 1.0 based on prior studies [ref. [1]]. The concordance between RT-PCR and IHC results was high. The Kappa coefficient and 95% confidence limits were 0.80 (0.57, 1.00). Click here for file

Additional file 2 **Selection of Candidate Genes profiled in the training set.** We selected 197 candidate genes from the published literature and microarray-based gene expression profiling experiments. The candidate genes included the 70-gene panel described in van't Veer et al, 104 genes further analyzed from Rosetta dataset (Dai et al), the Paik et al reported 16-gene panel (excluded the housekeeping genes from 21-gene panel), and 24 ER related genes. The 197 candidate genes and their accession number are listed. Click here for file

Additional file 3 **Normalization gene selection.** The expression level of 6 housekeeping genes (HSK) was determined on 150 breast cancer tissue samples. Gene stability was evaluated using the geNorm program [Ref. [2]] which relies on the principle that the expression ratio of two ideal reference genes would be identical in all samples, regardless of experimental conditions or cell type. The program calculates the gene stability measure (M) which is the average pair-wise variation for a gene compared with all other tested control gene. Genes with lower M values are more stable. The results show that PPIG, SLU7, and NUP214 were the most stable housekeeping genes in this sample set. Click here for file

Additional file 4 **mRNA Enhancement.** The RNAs used to profile the genes in the training set were enriched whereas the RNAs in the validation and tamoxifen treatment sets were used directly. To ensure that the profiling methods produced similar results, we compared the enriched and unenriched expression profile of 14 genes in 50 training set samples. Good correlation (R^2 ^= 0.9931) of gene expression levels was observed between the metastasis scores generated with the enriched vs. unenriched samples. Perfect agreement was obtained on risk category calls. Click here for file

Additional file 5 **Genes selected by supervised principal component procedure.** Table of genes selected by supervised principal component procedure. Click here for file

Additional file 6 **Coefficients of the 14 genes in the principle component.** Table and graphs of coefficients of the 14 genes in the principle component Click here for file

Additional file 7 **Algorithm for computing Metastasis Score (MS).** Algorithm for computing Metastasis Score (MS). The relative changes in gene expression are calculated by ΔΔCt method. Click here for file

Additional file 8 **Selection bias assessment.** Selection bias assessment in the training set and validation set. Click here for file

Additional file 9 **Clinical and pathological characteristics of patients from tamoxifen-treated set.** Table of clinical and pathological characteristics of patients from tamoxifen-treated set. Click here for file

Additional file 10 **Correlation between the Ki-67 LI and the MS and the Ki-67 mRNA level.** (a) Correlation of Ki-67 LI with MS, (b) Correlation of Ki-67 LI with Ki-67 mRNA level. R^2 ^= 0.30 and 0.18, were observed for Ki-67 LI versus MS, and Ki-67 LI versus Ki-67 mRNA level, respectively. Click here for file

Additional file 11 Ingenuity pathway analysis of prognostic 14-gene signature. The primary network identified below indicates the majority of the 14 genes of the signature are associated with known important tumor suppressors (TP53 and CDKN2A) and cell cycle control (CDC2). Nodes are displayed using various shapes that represent the functional class of the gene product (diamond-enzymes, ovals-transcription factors, triangles-kinase, circles-others). A solid line indicates a direct interaction while a dashed line indicates an indirect interaction. A line without an arrowhead indicates binding. Genes from 14-gene signature are highlighted in grey. Note that thirteen out of 14 genes of the signature are in the network. Click here for file

## References

[B1] van 't Veer LJ, Dai H, Vijver MJ van de, He YD, Hart AA, Mao M, Peterse HL, Kooy K van der, Marton MJ, Witteveen AT, Schreiber GJ, Kerkhoven RM, Roberts C, Linsley PS, Bernards R, Friend SH (2002). Gene expression profiling predicts clinical outcome of breast cancer. Nature.

[B2] van de Vijver MJ, He YD, van't Veer LJ, Dai H, Hart AA, Voskuil DW, Schreiber GJ, Peterse JL, Roberts C, Marton MJ, Parrish M, Atsma D, Witteveen A, Glas A, Delahaye L, Velde T van der, Bartelink H, Rodenhuis S, Rutgers ET, Friend SH, Bernards R (2002). A gene-expression signature as a predictor of survival in breast cancer. N Engl J Med.

[B3] Buyse M, Loi S, van't Veer L, Viale G, Delorenzi M, Glas AM, d'Assignies MS, Bergh J, Lidereau R, Ellis P, Harris A, Bogaerts J, Therasse P, Floore A, Amakrane M, Piette F, Rutgers E, Sotiriou C, Cardoso F, Piccart MJ, TRANSBIG Consortium (2006). Validation and clinical utility of a 70-gene prognostic signature for women with node-negative breast cancer. J Natl Cancer Inst.

[B4] Paik S, Shak S, Tang G, Kim C, Baker J, Cronin M, Baehner FL, Walker MG, Watson D, Park T, Hiller W, Fisher ER, Wickerham DL, Bryant J, Wolmark N (2004). A multigene assay to predict recurrence of tamoxifen-treated, node-negative breast cancer. N Engl J Med.

[B5] Paik S, Tang G, Shak S, Kim C, Baker J, Kim W, Cronin M, Baehner FL, Watson D, Bryant J, Costantino JP, Geyer CE, Wickerham DL, Wolmark N (2006). Gene expression and benefit of chemotherapy in women with node-negative, estrogen receptor-positive breast cancer. J Clin Oncol.

[B6] Goetz MP, Suman VJ, Ingle JN, Nibbe AM, Visscher DW, Reynolds CA, Lingle WL, Erlander M, Ma XJ, Sgroi DC, Perez EA, Couch FJ (2006). A two-gene expression ratio of homeobox 13 and interleukin-17B receptor for prediction of recurrence and survival in women receiving adjuvant tamoxifen. Clin Cancer Res.

[B7] Ma XJ, Hilsenbeck SG, Wang W, Ding L, Sgroi DC, Bender RA, Osborne CK, Allred DC, Erlander MG (2006). The HOXB13:IL17BR expression index is a prognostic factor in early-stage breast cancer. J Clin Oncol.

[B8] Jansen MP, Sieuwerts AM, Look MP, Ritstier K, Meijer-van Gelder ME, van Staveren IL, Klijn JG, Foekens JA, Berns EM (2007). HOXB13-to-IL17BR expression ratio is related with tumor aggressiveness and response to tamoxifen of recurrent breast cancer: a retrospective study. J Clin Oncol.

[B9] Wang Y, Klijn JG, Zhang Y, Sieuwerts AM, Look MP, Yang F, Talantov D, Timmermans M, Meijer-van Gelder ME, Yu J, Jatkoe T, Berns EM, Atkins D, Foekens JA (2005). Gene-expression profiles to predict distant metastasis of lymph-node-negative primary breast cancer. Lancet.

[B10] Foekens JA, Atkins D, Zhang Y, Sweep FC, Harbeck N, Paradiso A, Cufer T, Sieuwerts AM, Talantov D, Span PN, Tjan-Heijnen VC, Zito AF, Specht K, Hoefler H, Golouh R, Schittulli F, Schmitt M, Beex LV, Klijn JG, Wang Y (2006). Multicenter validation of a gene expression-based prognostic signature in lymph node-negative primary breast cancer. J Clin Oncol.

[B11] Desmedt C, Piette F, Loi S, Wang Lallemand F, Haibe-Kains B, Viale GY, Delorenzi M, Zhang Y, d'Assignies MS, Bergh J, Lidereau R, Ellis P, Harris AL, Klijn JG, Foekens JA, Cardoso F, Piccart MJ, Buyse M, Sotiriou C, TRANSBIG Consortium (2007). Strong time dependence of the 76-gene prognostic signature for node-negative breast cancer patients in the TRANSBIG multicenter independent validation series. Clin Cancer Res.

[B12] Chang HY, Nuyten DS, Sneddon JB, Hastie T, Tibshirani R, Sørlie T, Dai H, He YD, van't Veer LJ, Bartelink H, Rijn M van de, Brown PO, Vijver MJ van de (2005). Robustness, scalability, and integration of a wound-response gene expression signature in predicting breast cancer survival. Proc Natl Acad Sci USA.

[B13] Fan C, Oh DS, Wessels L, Weigelt B, Nuyten DS, Nobel AB, van't Veer LJ, Perou CM (2006). Concordance among gene-expression-based predictors for breast cancer. N Engl J Med.

[B14] Dai H, van't Veer L, Lamb J, He YD, Mao M, Fine BM, Bernards R, Vijver M van de, Deutsch P, Sachs A, Stoughton R, Friend S (2005). A cell proliferation signature is a marker of extremely poor outcome in a subpopulation of breast cancer patients. Cancer Res.

[B15] West M, Blanchette C, Dressman H, Huang E, Ishida S, Spang R, Zuzan H, Olson JA, Marks JR, Nevins JR (2001). Predicting the clinical status of human breast cancer by using gene expression profiles. Proc Natl Acad Sci USA.

[B16] World Health Organization, Geneva, Switzerland. (1982). Histological typing of breast tumours. Tumori.

[B17] Elston CW, Ellis IO (1991). Pathological prognostic factors in breast cancer. I. The value of histological grade in breast cancer: experience from a large study with long term follow-up. Histopathology.

[B18] Rogge L, Bianchi E, Biffi M, Bono E, Chang SY, Alexander H, Santini C, Ferrari G, Sinigaglia L, Seiler M, Neeb M, Mous J, Sinigaglia F, Certa U (2000). Transcript imaging of the development of human T helper cells using oligonucleotide arrays. Nat Genet.

[B19] Livak KJ, Schmittgen TD (2001). Analysis of relative gene expression data using real-time quantitative PCR and the 2(-Delta Delta C(T)) method. Methods.

[B20] Troyanskaya O, Cantor M, Sherlock G, Brown P, Hastien T, Tibshirani R, Botstein D, Altman RB (2001). Missing value estimation methods for DNA microarrays. Bioinformatics.

[B21] Bair E, Tibshirani R (2004). Semi-supervised methods to predict patient survival from gene expression data. PloS Biology.

[B22] Bair E, Hastie T, Debashis P, Tibshirani R (2006). Prediction by supervised principal components. J Am Stat Assoc.

[B23] Hastie TJ, Narasimhan B, Tibshirani RJ (2005). PAM -Prediction Analysis of Microarrays. Software online.

[B24] Cox DR (1972). Regression models and life tables (with discussion). J Royl Stat Soc.

[B25] Tibshirani R (1996). Regression shrinkage and selection via the lasso. J Roy Stat Soc B.

[B26] Kaplan EL, Meier P (1958). Nonparametric estimation from incomplete observations. J Am Stat Assoc.

[B27] Collett D (1994). Modeling Survival Data in Medical Research.

[B28] Harrell F (2001). Regression Modeling Strategies: with applications to linear models, logistic regression, and survival analysis.

[B29] (2001). SAS software, version 9.1.

[B30] (2007). R: A Language and Environment for Statistical Computing.

[B31] Heagerty PJ, Lumley T, Pepe MS (2000). Time-Dependent ROC Curves for Censored Survival Data and a Diagnostic Marker. Biometrics.

[B32] Akritas MG (1994). Nearest neighbor estimation of a bivariate distribution under random censoring. Annals of Statistics.

[B33] Efron B, Tibshirani RJ (1993). An Introduction to the Bootstrap.

[B34] Flanagan MB, Dabbs DJ, Brufsky AM, Beriwal S, Bhargava R (2008). Histopathologic variables predict Oncotype DXtrade mark Recurrence Score. Mod Pathol.

[B35] Adjuvant! Online. http://www.adjuvantonline.com/index.jsp.

[B36] Edén P, Ritz C, Rose C, Fernö M, Peterson C (2004). "Good Old" clinical markers have similar power in breast cancer prognosis as microarray gene expression profilers. Eur J Cancer.

[B37] Vincent-Salomon A, Rousseau A, Jouve M, Beuzeboc P, Sigal-Zafrani B, Fréneaux P, Rosty C, Nos C, Campana F, Klijanienko J, Al Ghuzlan A, Sastre-Garau X, Breast Cancer Study Group (2004). Proliferation markers predictive of the pathological response and disease outcome of patients with breast carcinomas treated by anthracycline-based preoperative chemotherapy. Eur J Cancer.

[B38] Burcombe R, Wilson GD, Dowsett M, Khan I, Richman PI, Daley F, Detre S, Makris A (2006). Evaluation of Ki-67 proliferation and apoptotic index before, during and after neoadjuvant chemotherapy for primary breast cancer. Breast Cancer Res.

[B39] Colozza M, Azambuja E, Cardoso F, Sotiriou C, Larsimont D, Piccart MJ (2005). Proliferative markers as prognostic and predictive tools in early breast cancer: where are we now?. Ann Oncol.

[B40] Dowsett M, Smith IE, Ebbs SR, Dixon JM, Skene A, A'Hern R, Salter J, Detre S, Hills M, Walsh G, IMPACT Trialists Group (2007). Prognostic value of Ki67 expression after short-term presurgical endocrine therapy for primary breast cancer. J Natl Cancer Inst.

[B41] Miller WR, Dixon JM, Macfarlane L, Cameron D, Anderson TJ (2003). Pathological features of breast cancer response following neoadjuvant treatment with either letrozole or tamoxifen. Eur J Cancer.

[B42] Potemski P, Pluciennik E, Bednarek AK, Kusinska R, Kubiak R, Jesionek-Kupnicka D, Watala C, Kordek R (2006). Ki-67 expression in operable breast cancer: a comparative study of immunostaining and a real-time RT-PCR assay. Pathol Res Pract.

[B43] Tan PH, Bay BH, Yip G, Selvarajan S, Tan P, Wu J, Lee CH, Li KB (2005). Immunohistochemical detection of Ki67 in breast cancer correlates with transcriptional regulation of genes related to apoptosis and cell death. Mod Patho.

[B44] de Azambuja E, Cardoso F, de Castro G Jr, Colozza M, Mano MS, Durbecq V, Sotiriou C, Larsimont D, Piccart-Gebhart MJ, Paesmans M (2007). Ki-67 as prognostic marker in early breast cancer: a meta-analysis of published studies involving 12,155 patients. Br J Cancer.

[B45] Whitfield ML, George LK, Grant GD, Perou CM (2006). Common markers of proliferation. Nat Rev Cancer.

[B46] Miller LD, Smeds J, George J, Vega VB, Vergara L, Ploner A, Pawitan Y, Hall P, Klaar S, Liu ET, Bergh J (2005). An expression signature for p53 status in human breast cancer predicts mutation status, transcriptional effects, and patient survival. Proc Natl Acad Sci USA.

[B47] Pai SI, Lin YY, Macaes B, Meneshian A, Hung CF, Wu TC (2006). Prospects of RNA interference therapy for cancer. Gene Ther.

[B48] Akinc A, Zumbuehl A, Goldberg M, Leshchiner ES, Busini V, Hossain N, Bacallado SA, Nguyen DN, Fuller J, Alvarez R, Borodovsky A, Borland T, Constien R, de Fougerolles A, Dorkin JR, Narayanannair Jayaprakash K, Jayaraman M, John M, Koteliansky V, Manoharan M, Nechev L, Qin J, Racie T, Raitcheva D, Rajeev KG, Sah DW, Soutschek J, Toudjarska I, Vornlocher HP, Zimmermann TS, Langer R, Anderson DG (2008). A combinatorial library of lipid-like materials for delivery of RNAi therapeutics. Nat Biotechnol.

[B49] Devi GR (2006). siRNA-based approaches in cancer therapy. Cancer Gene Ther.

[B50] Hayes DF (2005). Prognostic and predictive factors revisited. Breast.

[B51] Esteva FJ, Sahin AA, Cristofanilli M, Coombes K, Lee SJ, Baker J, Cronin M, Walker M, Watson D, Shak S, Hortobagyi GN (2005). Prognostic role of a multigene reverse transcriptase-PCR assay in patients with node-negative breast cancer not receiving adjuvant systemic therapy. Clin Cancer Res.

[B52] Carey LA, Perou CM, Livasy CA, Dressler LG, Cowan D, Conway K, Karaca G, Troester MA, Tse CK, Edmiston S, Deming SL, Geradts J, Cheang MC, Nielsen TO, Moorman PG, Earp HS, Millikan RC (2006). Race, breast cancer subtypes, and survival in the Carolina Breast Cancer Study. JAMA.

