# Supplementary material for: Risk estimation of distant metastasis in node-negative, estrogen receptor-positive breast cancer patients using an RT-PCR based prognostic expression signature
Source: BMC Cancer. 2008 Nov 21;8:339. doi: 10.1186/1471-2407-8-339 (PMC2631011; doi:10.1186/1471-2407-8-339)
Supplement: Additional file 1 — Concordance for ER status by immunhistochemistry and RT-PCR. We evaluated the concordance of ER status determined by RT-PCR and IHC for the Guy's untreated patients. Of the 287 patients, 97.6% and 96.9% were ER-positive by IHC and RT-PCR assay, respectively. The cutoff point for ER positivity of RT-PCR assay was prespecified as 1.0 based on prior studies [ref. [1]]. The concordance between RT-PCR and IHC results was high. The Kappa coefficient and 95% confidence limits were 0.80 (0.57, 1.00). [file 1471-2407-8-339-S1.pdf]

Additional file 1
File format: DOC
Title: Concordance for ER status by immunhistochemistry and RT-PCR
Description: We evaluated the concordance of ER status determined by RT-PCR and IHC for the Guy's untreated patients. Of the 287 patients, 97.6% and 96.9% were ER-positive by IHC and RT-PCR assay, respectively. The cutoff point for ER positivity of RT-PCR assay was prespecified as 1.0 based on prior studies [ref. 1]. The concordance between RT-PCR and IHC results was high. The Kappa coefficient and 95% confidence limits were 0.80 (0.57, 1.00).

Correlation between IHC and RT-PCR measurements for ER

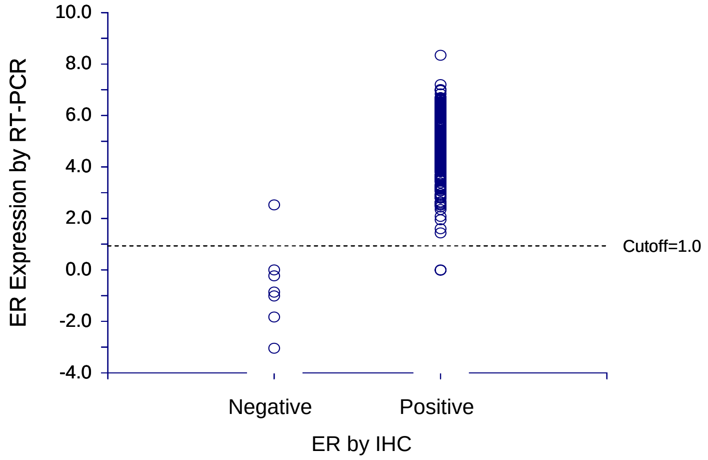

Ref. 1. Iverson A, Gillett C, Cane P, Santini C, Vess T, Kam-Morgan L, Wang A, Eisenberg M, Rowland C, Hessling J, Broder S, Sninsky J, Tutt A, Anderson S, Chang S-Y: A Novel Single-Tube Quantitative Assay for mRNA Levels of Hormonal and Growth Factor Receptors in Breast Cancer Formalin-fixed, Paraffin-embedded Sections. (J Mol Diagn, in press)
