# Supplementary material for: Risk estimation of distant metastasis in node-negative, estrogen receptor-positive breast cancer patients using an RT-PCR based prognostic expression signature
Source: BMC Cancer. 2008 Nov 21;8:339. doi: 10.1186/1471-2407-8-339 (PMC2631011; doi:10.1186/1471-2407-8-339)
Supplement: Additional file 2 — Selection of Candidate Genes profiled in the training set. We selected 197 candidate genes from the published literature and microarray-based gene expression profiling experiments. The candidate genes included the 70-gene panel described in van't Veer et al, 104 genes further analyzed from Rosetta dataset (Dai et al), the Paik et al reported 16-gene panel (excluded the housekeeping genes from 21-gene panel), and 24 ER related genes. The 197 candidate genes and their accession number are listed. [file 1471-2407-8-339-S2.pdf]

Additional file 2
File format: DOC
Title: Selection of Candidate Genes profiled in the training set
Description: We selected 197 candidate genes from the published literature and microarray-based gene expression profiling experiments. The candidate genes included the 70-gene panel described in van't Veer et al, 104 genes further analyzed from Rosetta dataset (Dai et al), the Paik et al reported 16-gene panel (excluded the housekeeping genes from 21-gene panel), and 24 ER related genes. The 197 candidate genes and their accession number are listed below.

**LL_Symbol**	**Accession Number**
AARS	NM_001605
ABCB6	NM_005689
ACBD4	NM_024722
ADAM8	NM_001109
ADM	NM_001124
AI224578	CONTIG40831RC
AK026372	AK026372
AK123483	CONTIG63649RC
AKAP2	NM_007203
ALDH4A1	NM_003748
ALDOC	NM_005165
AP2B1	NM_001282
APOBEC3B	NM_004900
AR	NM_000044
AURKA	NM_003600
AYTL2	NM_024830
BAG1	NM_004323
BBC3	NM_014417
BC039414	BC039414
BCL2	NM_000633
BIRC5	NM_001168
BLM	NM_000057
BTD	NM_000060
BUB1	NM_004336
C15orf42	NM_152259
C16orf61	NM_020188
C20orf46	NM_018354
C9orf30	NM_080655
C9orf46	NM_018465
CAPZB	NM_004930
CCNB1	NM_031966
CCNB2	NM_004701
CCNE2	NM_004702
CD68	NM_001251
CDC42BPA	NM_003607
CDC6	NM_001254
CDCA3	NM_031299
CDCA7	NM_031942
CENPA	NM_001809
CENPN	NM_018455
COL14A1	NM_021110

**LL_Symbol**	**Accession Number**
KPNA2	NM_002266
LAGE3	NM_006014
LDHA	NM_005566
LIN9	NM_173083
LOC124220	NM_145252
LOC286052	AK095104
LRRC17	NM_005824
MAD2L1	NM_002358
MAPT	NM_005910
MASTL	NM_032844
MCM6	NM_005915
MELK	NM_014791
MKI67	NM_002417
MLF1IP	NM_024629
MMP3	NM_002422
MMP9	NM_004994
MS4A7	NM_021201
MTDH	NM_178812
MYB	NM_005371
MYBL2	NM_002466
MYSM1	XM_055481
NAT1	NM_000662
NDC80	NM_006101
NDRG1	NM_006096
NME1	NM_000269
NMU	NM_006681
NTN4	NM_021229
NUSAP1	NM_016359
ORC6L	NM_014321
OXCT1	NM_000436
P2RX4	NM_002560
P4HA1	NM_000917
PARD6B	XM_030559
PECI	NM_006117
PERLD1	NM_033419
PFKFB4	NM_004567
PFKP	NM_002627
PGK1	NM_000291
PGR	NM_000926
PHLDA2	NM_003311
PITRM1	NM_014889

COL4A2	NM_001846	PKMYT1	NM_004203
CONTIG46218RC	CONTIG46218RC	PPIE	NM_006112
CSTA	NM_005213	PQLC2	NM_017765
CTSC	NM_001814	PRC1	NM_003981
CTSD	NM_001909	PRR11	NM_018304
CTSL2	NM_001333	PSMD7	NM_002811
CX3CL1	NM_002996	PTTG1	NM_004219
DCK	NM_000788	QSOX2	NM_181701
DEPDC1B	NM_018369	RAB6B	NM_016577
DHX58	NM_024119	RACGAP1	NM_013277
DIAPH3	NM_030932	RARRES1	NM_002888
DTL	NM_016448	RASSF7	NM_003475
E2F1	NM_004168	RECQL5	NM_001003715
EBF4	XM_044921	RFC4	NM_002916
ECT2	NM_018098	RHBDF2	NM_024599
EGLN1	NM_022051	RPL27	NM_000988
EIF3S3	NM_003756	RRM2	NM_001034
ENO1	NM_001428	RTN4RL1	XM_091486
ERBB2	NM_004448	RUNDC1	NM_173079
ESM1	NM_007036	S100A9	NM_002965
ESPL1	NM_012291	SCD	NM_005063
ESR1	NM_000125	SCUBE2	NM_020974
EXOC7	XM_036173	SERF1A	NM_021967
EXT1	NM_000127	SERPINB5	NM_002639
FADS1	NM_013402	SESN3	NM_144665
FBXO31	NM_024735	SF3B3	NM_012426
FGF18	NM_003862	SLC16A3	NM_004207
FLNA	NM_001456	SLC2A1	NM_006516
FLT1	NM_002019	SLC2A3	NM_006931
FOXA1	NM_000181	SLC39A6	NM_012319
FST	NM_006350	SNRPA1	NM_003090
GAPDH	NM_002046	SPAG5	NM_006461
GATA3	NM_002051	SPOCD1	NM_144569
GCLM	NM_002061	SPP1	NM_000582
GINS2	NM_016095	SRD5SA2	NM_024592
GMPS	NM_003875	STK32B	NM_018401
GNAZ	NM_002073	SUV39H2	NM_024670
GNG12	NM_018841	SYNGR2	NM_004710
GPR126	NM_152339	TFF1	NM_003225
GPR180	AK024059	TFF3	NM_003226
GRB7	NM_005310	TGFB3	NM_003239
GRHL2	NM_024915	TK1	NM_003258
GSTM1	NM_000561	TLCD1	NM_138463
GSTM3	NM_000849	TMC6	NM_007267
H2AFZ	NM_002106	TMEM158	NM_015444
H30384	CONTIG47405RC	TMEM176A	NM_018487
HN1	NM_016185	TPI1	NM_000365
HOOK1	AK027250	TSPYL5	NM_033512
HPN	NM_002151	TUBB4	NM_006086
HRASLS	NM_020386	UBE2C	NM_007019
HSPA5	NM_005347	UBE2S	NM_014501
IGFBP5	NM_000599	UCHL5	NM_015984
IL8	NM_000584	VEGFA	NM_003376

INPP4B	NM_003866
ITGA5	NM_002205
KIAA0125	NM_014792
KIF23	NM_004856
KIF2C	NM_006845

WISP1	NM_003882
YWHAZ	NM_003406
ZNF367	NM_153695
ZNF385B	NM_001113397
