# Supplementary material for: Risk estimation of distant metastasis in node-negative, estrogen receptor-positive breast cancer patients using an RT-PCR based prognostic expression signature
Source: BMC Cancer. 2008 Nov 21;8:339. doi: 10.1186/1471-2407-8-339 (PMC2631011; doi:10.1186/1471-2407-8-339)
Supplement: Additional file 3 — Normalization gene selection. The expression level of 6 housekeeping genes (HSK) was determined on 150 breast cancer tissue samples. Gene stability was evaluated using the geNorm program [Ref. [2]] which relies on the principle that the expression ratio of two ideal reference genes would be identical in all samples, regardless of experimental conditions or cell type. The program calculates the gene stability measure (M) which is the average pair-wise variation for a gene compared with all other tested control gene. Genes with lower M values are more stable. The results show that PPIG, SLU7, and NUP214 were the most stable housekeeping genes in this sample set. [file 1471-2407-8-339-S3.pdf]

Additional file 3
File format: DOC
Title: Normalization gene selection
Description: The expression level of 6 housekeeping genes (HSK) was determined on 150 breast cancer tissue samples. Gene stability was evaluated using the geNorm program [Ref. 2] which relies on the principle that the expression ratio of two ideal reference genes would be identical in all samples, regardless of experimental conditions or cell type. The program calculates the gene stability measure (M) which is the average pair-wise variation for a gene compared with all other tested control gene. Genes with lower M values are more stable. The results show that PPIG, SLU7, and NUP214 were the most stable housekeeping genes in this sample set.

**Gene**	**M value**
PPIG	0.8046
SLU7	0.8741
NUP214	0.8886
PPP1CA	1.0256
TERF2	1.1907
EEF1A1	1.1994

The expression stability of these 3 genes was confirmed on 138 breast cancer FFPE samples. All three genes were selected for use in normalizing RT-PCR results for all the genes used in this study.

**Gene**	**M value**
PPIG	0.5697
NUP214	0.5520
SLU7	0.6075

Ref. 2. Vandesompele J, De Preter K, Pattyn F, Poppe B, Van Roy N, De Paepe A, Speleman F: Accurate normalization of real-time quantitative RT-PCR data by geometric averaging of multiple internal control genes. Genome Biol 2002, 7:RESEARCH0034.
