# Supplementary material for: Risk estimation of distant metastasis in node-negative, estrogen receptor-positive breast cancer patients using an RT-PCR based prognostic expression signature
Source: BMC Cancer. 2008 Nov 21;8:339. doi: 10.1186/1471-2407-8-339 (PMC2631011; doi:10.1186/1471-2407-8-339)

Additional file 4
File format: DOC
Title: mRNA Enhancement
Description: The RNAs used to profile the genes in the training set were enriched whereas the RNAs in the validation and tamoxifen treatment sets were used directly. To ensure that the profiling methods produced similar results, we compared the enriched and unenriched expression profile of 14 genes in 50 training set samples. Good correlation (R2=0.9931) of gene expression levels was observed between the metastasis scores generated with the enriched vs. unenriched samples. Perfect agreement was obtained on risk category calls.

Concordance of Enriched vs. Unenriched Samples

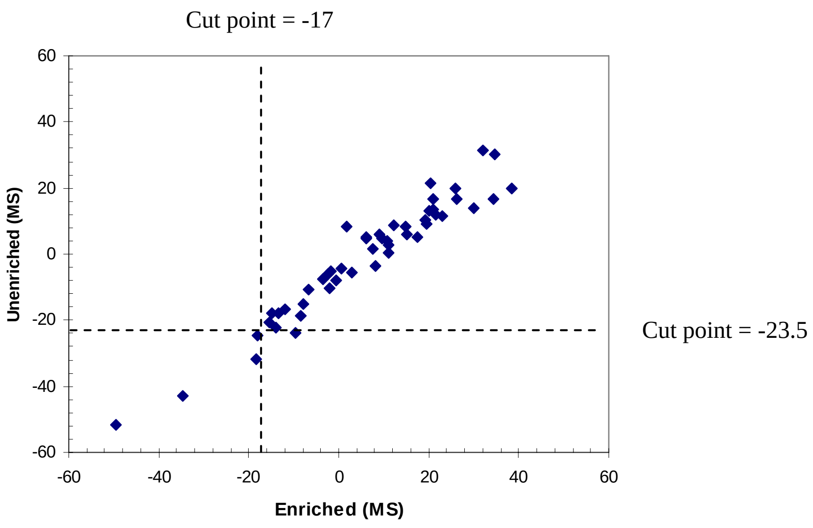

Supplement: Additional file 4 — mRNA Enhancement. The RNAs used to profile the genes in the training set were enriched whereas the RNAs in the validation and tamoxifen treatment sets were used directly. To ensure that the profiling methods produced similar results, we compared the enriched and unenriched expression profile of 14 genes in 50 training set samples. Good correlation (R2 = 0.9931) of gene expression levels was observed between the metastasis scores generated with the enriched vs. unenriched samples. Perfect agreement was obtained on risk category calls. [file 1471-2407-8-339-S4.pdf]
