# Supplementary material for: Risk estimation of distant metastasis in node-negative, estrogen receptor-positive breast cancer patients using an RT-PCR based prognostic expression signature
Source: BMC Cancer. 2008 Nov 21;8:339. doi: 10.1186/1471-2407-8-339 (PMC2631011; doi:10.1186/1471-2407-8-339)
Supplement: Additional file 5 — Genes selected by supervised principal component procedure. Table of genes selected by supervised principal component procedure. [file 1471-2407-8-339-S5.pdf]

Additional file 5
File format: DOC
Title: Genes selected by supervised principal component procedure
Description:

Gene	p-value	Hazard Ratio	Lower 95% CL	Upper 95% CL
MELK*	<.0001	2.32	1.55	3.46
UBE2C	0.0004	2.17	1.41	3.33
DIAPH3*	0.0002	2.04	1.41	2.95
PKMYT1*	0.0001	2.01	1.40	2.88
CONTIG63649RC	0.0003	1.89	1.34	2.66
NUSAP1	0.0026	1.83	1.24	2.71
MYBL2*	0.0005	1.82	1.30	2.55
CENPA*	0.0005	1.81	1.29	2.52
ESPL1	0.0027	1.79	1.23	2.62
CONTIG46218RC	0.0029	1.79	1.22	2.61
SPOCD1	0.0035	1.74	1.20	2.53
FBXO31	0.0012	1.74	1.24	2.43
TK1*	0.0061	1.71	1.17	2.51
MMP9	0.0041	1.71	1.18	2.47
DEPDC1B	0.0033	1.70	1.19	2.43
PRR11*	0.0013	1.69	1.23	2.34
UBE2S*	0.0011	1.69	1.23	2.31
RFC4*	0.0010	1.68	1.23	2.28
BUB1*	0.0158	1.66	1.10	2.52
CCNE2	0.0080	1.65	1.14	2.39
SERF1A	0.0006	1.61	1.23	2.12
RHBDF2	0.0048	1.59	1.15	2.19
ORC6L*	0.0037	1.58	1.16	2.16
RACGAP1*	0.0115	1.56	1.10	2.19
KIF2C	0.0162	1.55	1.08	2.23
CENPN	0.0190	1.52	1.07	2.16
CCNB1*	0.0305	1.50	1.04	2.16
DTL	0.0246	1.49	1.05	2.10
EBF4	0.0253	1.47	1.05	2.07
DC13*	0.0136	1.46	1.08	1.98
QSOX2	0.0276	1.42	1.04	1.94
SLC2A3	0.0219	1.41	1.05	1.90
STK32B	0.0364	0.73	0.54	0.98
NAT1	0.0136	0.70	0.53	0.93
GSTM1	0.0324	0.67	0.46	0.97
NTN4	0.0049	0.64	0.46	0.87
ZNF533	0.0036	0.59	0.42	0.84

*genes in the14-gene model from the 37-gene model reduced by LASSO via LARS
