# Supplementary material for: Risk estimation of distant metastasis in node-negative, estrogen receptor-positive breast cancer patients using an RT-PCR based prognostic expression signature
Source: BMC Cancer. 2008 Nov 21;8:339. doi: 10.1186/1471-2407-8-339 (PMC2631011; doi:10.1186/1471-2407-8-339)
Supplement: Additional file 6 — Coefficients of the 14 genes in the principle component. Table and graphs of coefficients of the 14 genes in the principle component [file 1471-2407-8-339-S6.pdf]

Additional file 6
File format: DOC
Title: Coefficients of the 14 genes in the principle component
Description:

Gene	ai
DC13	0.22
DIAPH3	0.23
CCNB1	0.23
RFC4	0.25
PRR11	0.26
UBE2S	0.27
TK1	0.27
BUB1	0.27
ORC6L	0.28
CENPA	0.29
MELK	0.29
MYBL2	0.29
PKMYT1	0.29
RACGAP1	0.29

ai = loadings on the first principal component

Correlation of MS calculated by using regression coefficient versus sum of 14 ΔΔCt

Training set

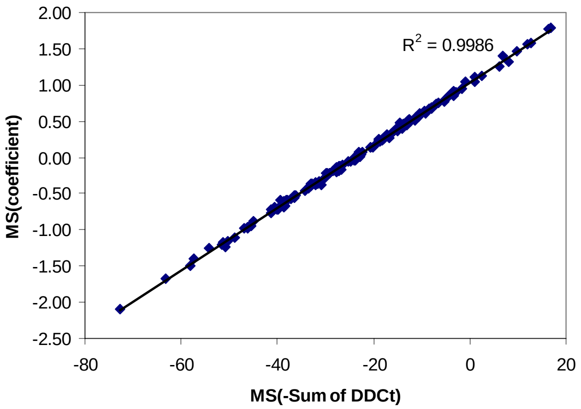

Validation set

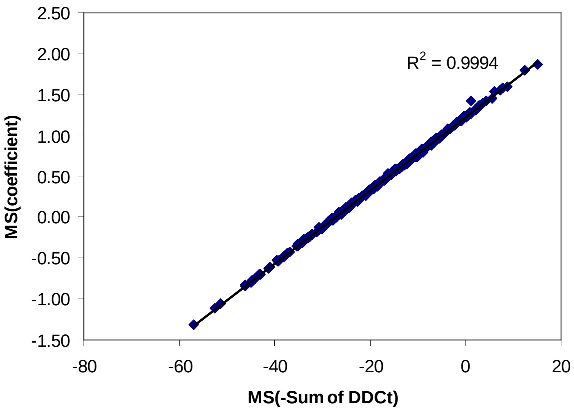

Near perfect correlations, $R^2 = 0.9986$ and 0.9994, were observed for the MS calculated by applying regression coefficient versus the sum of the 14 ΔΔCt measurements for each subject in the training and validation set, respectively.
