# Supplementary material for: Risk estimation of distant metastasis in node-negative, estrogen receptor-positive breast cancer patients using an RT-PCR based prognostic expression signature
Source: BMC Cancer. 2008 Nov 21;8:339. doi: 10.1186/1471-2407-8-339 (PMC2631011; doi:10.1186/1471-2407-8-339)
Supplement: Additional file 7 — Algorithm for computing Metastasis Score (MS). Algorithm for computing Metastasis Score (MS). The relative changes in gene expression are calculated by ΔΔCt method. [file 1471-2407-8-339-S7.pdf]

Additional file 7
File format: DOC
Title: Algorithm for computing Metastasis Score (MS)
Description: The relative changes in gene expression are calculated by $\Delta\Delta$Ct method. The level of mRNA of each of the 14 profiled genes is defined as:

$$\Delta Ct_{test} = (Ct_{GOI} - Ct_{EC})_{test\ RNA}$$

$$\Delta Ct_{ref} = (Ct_{GOI} - Ct_{EC})_{ref\ RNA}$$

$$Gi = \Delta\Delta Ct = \Delta Ct_{test} - \Delta Ct_{ref}$$

where GOI = gene of interest (each of 14 signature genes), EC = endogenous control , test RNA = RNA obtained from the patient sample, and ref RNA = a calibrator reference RNA.. The expression level (Ct) measured by RT-PCR of each signature gene is first normalized to the average of three endogenous control genes resulting ΔCt_{test} and ΔCt_{ref} for test RNA and reference RNA, respectively. Then, the difference ($\Delta\Delta$Ct) between ΔCt_{test} and ΔCt_{ref} is the relative expression level of the gene of interest.

Since the coefficients of the 14 genes were of similar magnitude (Table S.8), a summary score was calculated as the sum of the 14 $\Delta\Delta$Ct measurements for each subject, and since lower values of the score were associated with higher probability of metastasis, the final MS for each subject is defined as the negative of the summary score.

The $\Delta\Delta$Ct value, obtained in gene expression profiling for each of the 14 signature genes, can be used in the following formula to generate a MS:

$$MS = -\left[\sum_{i=1}^{14} Gi\right]$$

in which Gi represents the expression level of each gene (i) of the 14-gene prognostic signature. The value of Gi is the $\Delta\Delta$Ct obtained in expression profiling described above. The median MS observed in the training set was used as a cutoff to determine categories of high and low prognostic risk.
