# Supplementary material for: Risk estimation of distant metastasis in node-negative, estrogen receptor-positive breast cancer patients using an RT-PCR based prognostic expression signature
Source: BMC Cancer. 2008 Nov 21;8:339. doi: 10.1186/1471-2407-8-339 (PMC2631011; doi:10.1186/1471-2407-8-339)
Supplement: Additional file 8 — Selection bias assessment. Selection bias assessment in the training set and validation set. [file 1471-2407-8-339-S8.pdf]

Additional file 8
File format: DOC
Title: Selection bias assessment
Description:

Selection bias assessment in the training set

Characteristics	Included n=142 n (%)	Excluded n=67 n (%)	Selection bias P-value (two-sided)
Age			0.6987*
≤ 55 yrs	143 (51.3)	22 (32.8)	
> 55 yrs	136 (48.8)	45 (67.2)	
Missing	0	0	
Median	64 yrs (SD 12.6)	63.7 yrs (SD 13.7)	
Min. - Max.	31 - 89 yrs	34 - 95 yrs	
Tumor diameter			0.3909*
≤ 2 cm	126 (94.0)	60 (93.8)	
> 2 cm	8 (6.0)	4 (6.3)	
Missing	8	3	
Median	1.2 cm (SD 0.50)	1.23 cm (SD 0.47)	
Min. - Max.	0.3 - 2.9 cm	0.3 - 2.6 cm	
Tumor grade			0.0247**
Grade 1	74 (53.2)	43 (68.3)	
Grade 2	61 (43.9)	16 (25.4)	
Grade 3	4 (2.9)	4 (6.4)	
Missing	3	4	

*Wilcoxon rank sum test; **Fisher's exact test

No significant differences were found between the included and excluded subjects with respect to age or tumor size. However, tumor grade tended to be higher among the included subjects than the excluded subjects.

Selection bias assessment in the validation set

Characteristics	Included n=279 n (%)	Excluded n=118 n (%)	Selection bias P-value (two-sided)
Age			0.4679*
≤ 55 yrs	143 (51.3)	22 (32.8)	
> 55 yrs	136 (48.8)	45 (67.2)	
Missing	0	0	
Median	55 yrs (SD 11.7)	58 yrs (SD 12.1)	
Min. - Max.	29 - 87 yrs	27 - 86 yrs	
Tumor diameter			0.0235*
≤ 2 cm	168 (60.2)	82 (69.5)	
> 2 cm	111 (39.8)	36 (30.5)	
Median	2 cm (SD 0.85)	2 cm (SD 0.87)	
Min. - Max.	0.0# - 3.0 cm	0.0# - 3.0 cm	
Tumor grade			0.179**
Grade 1	60 (21.5)	28 (28.9)	
Grade 2	166 (59.5)	57 (58.8)	
Grade 3	53 (19.0)	12 (12.4)	
Missing	0	21	

*Wilcoxon rank sum test; **Fisher's exact test; # tumors were impalpable

No significant differences between included and excluded patients were found with respect to age or tumor grade. We found a somewhat larger proportion of patients with tumor size greater than 2 cm among the included patients than among the excluded patients.
