# Supplementary material for: Risk estimation of distant metastasis in node-negative, estrogen receptor-positive breast cancer patients using an RT-PCR based prognostic expression signature
Source: BMC Cancer. 2008 Nov 21;8:339. doi: 10.1186/1471-2407-8-339 (PMC2631011; doi:10.1186/1471-2407-8-339)
Supplement: Additional file 9 — Clinical and pathological characteristics of patients from tamoxifen-treated set. Table of clinical and pathological characteristics of patients from tamoxifen-treated set. [file 1471-2407-8-339-S9.pdf]

Additional file 9
File format: DOC
Title: Clinical and pathological characteristics of patients from tamoxifen-treated set
Description:

**Characteristics**	**Tam-Treated n=45 n (%)**
Age	
≤ 55 yrs	4 (26.7)
> 55 yrs	11 (73.3)
Missing	30
Median	57 yrs (SD 13.8)
Min. - Max.	26 - 80 yrs
Tumor diameter	
≤ 2 cm	45 (100)
Tumor grade	
Grade 1	7 (17.5)
Grade 2	24 (60.0)
Grade 3	9 (22.5)
Missing	5
Stage	
I	45 (100)
Distant recurrence	
Yes	8 (17.8)
No	37 (82.2)
Median follow up	5.8 yrs
