# Supplementary material for: Risk estimation of distant metastasis in node-negative, estrogen receptor-positive breast cancer patients using an RT-PCR based prognostic expression signature
Source: BMC Cancer. 2008 Nov 21;8:339. doi: 10.1186/1471-2407-8-339 (PMC2631011; doi:10.1186/1471-2407-8-339)
Supplement: Additional file 10 — Correlation between the Ki-67 LI and the MS and the Ki-67 mRNA level. (a) Correlation of Ki-67 LI with MS, (b) Correlation of Ki-67 LI with Ki-67 mRNA level. R2 = 0.30 and 0.18, were observed for Ki-67 LI versus MS, and Ki-67 LI versus Ki-67 mRNA level, respectively. [file 1471-2407-8-339-S10.pdf]

Additional file 10
File format: DOC
Title: Correlation between the Ki-67 LI and the MS and the Ki-67 mRNA level
Description: (a) Correlation of Ki-67 LI with MS, (b) Correlation of Ki-67 LI with Ki-67 mRNA level

(a)

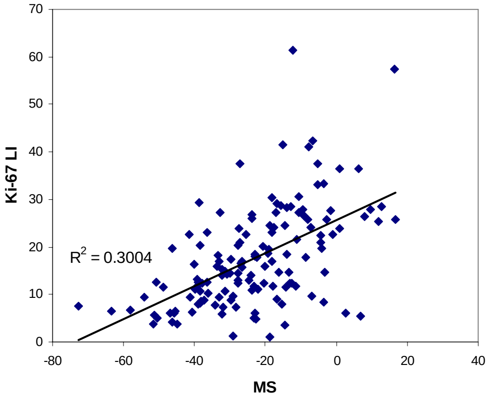

(b)

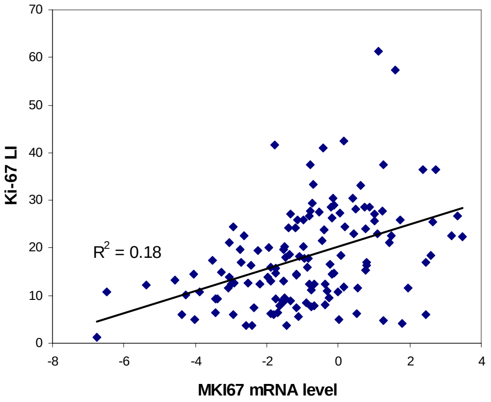

R2 = 0.30 and 0.18, were observed for Ki-67 LI versus MS, and Ki-67 LI versus Ki-67 mRNA level, respectively.
