# Supplementary material for: Risk estimation of distant metastasis in node-negative, estrogen receptor-positive breast cancer patients using an RT-PCR based prognostic expression signature
Source: BMC Cancer. 2008 Nov 21;8:339. doi: 10.1186/1471-2407-8-339 (PMC2631011; doi:10.1186/1471-2407-8-339)

Additional file 11
File format: DOC
Title: Ingenuity pathway analysis of prognostic 14-gene signature
Description: The primary network identified below indicates the majority of the 14 genes of the signature are associated with known important tumor suppressors (TP53 and CDKN2A) and cell cycle control (CDC2). Nodes are displayed using various shapes that represent the functional class of the gene product (diamond-enzymes, ovals-transcription factors, triangles-kinase, circles-others). A solid line indicates a direct interaction while a dashed line indicates an indirect interaction. A line without an arrowhead indicates binding. Genes from 14-gene signature are highlighted in grey. Note that thirteen out of 14 genes of the signature are in the network.

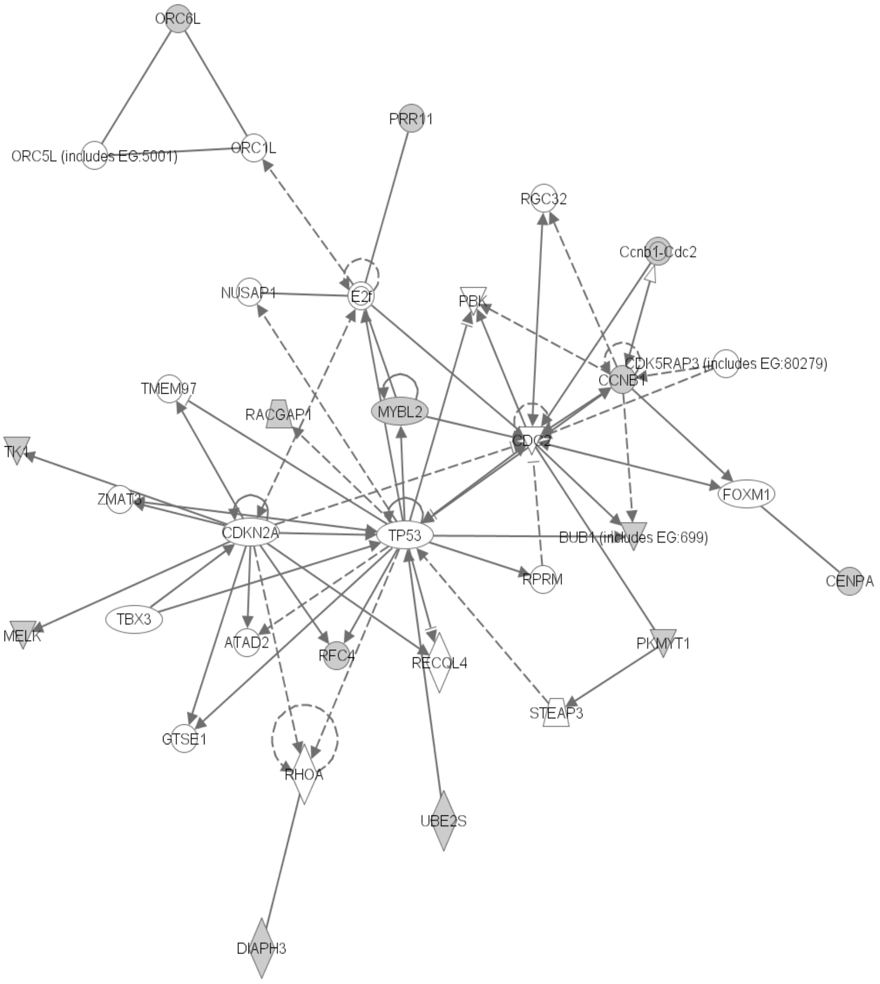

Supplement: Additional file 11 — Ingenuity pathway analysis of prognostic 14-gene signature. The primary network identified below indicates the majority of the 14 genes of the signature are associated with known important tumor suppressors (TP53 and CDKN2A) and cell cycle control (CDC2). Nodes are displayed using various shapes that represent the functional class of the gene product (diamond-enzymes, ovals-transcription factors, triangles-kinase, circles-others). A solid line indicates a direct interaction while a dashed line indicates an indirect interaction. A line without an arrowhead indicates binding. Genes from 14-gene signature are highlighted in grey. Note that thirteen out of 14 genes of the signature are in the network. [file 1471-2407-8-339-S11.pdf]
